# Mitochondrial function in peripheral blood cells across the human lifespan

**DOI:** 10.1038/s41514-023-00130-4

**Published:** 2024-02-07

**Authors:** Johannes K. Ehinger, Emil Westerlund, Eleonor Åsander Frostner, Michael Karlsson, Gesine Paul, Fredrik Sjövall, Eskil Elmér

**Affiliations:** 1 Otorhinolaryngology, Head and Neck Surgery, Department of Clinical Sciences Lund, Lund University, Lund, Sweden; 2 Mitochondrial Medicine, Department of Clinical Sciences Lund, Lund University, Lund, Sweden; 3 Otorhinolaryngology, Head and Neck Surgery, Skåne University Hospital, Lund, Sweden; 4 Emergency Department, Kungälv Hospital, Kungälv, Sweden; 5 Department of Neurosurgery, Rigshospitalet, Copenhagen, Denmark; 6 Translational Neurology Group and Wallenberg Center for Molecular Medicine, Department of Clinical Sciences Lund, Lund University, Lund, Sweden; 7 Department of Intensive- and Perioperative Care, Skåne University Hospital, Malmö, Sweden; 8 Clinical Neurophysiology, Medical Imaging and Physiology, Skåne University Hospital, Lund, Sweden

**Keywords:** Senescence, Ageing

## Abstract

Mitochondrial dysfunction is considered a hallmark of aging. Up to now, a gradual decline of mitochondrial respiration with advancing age has mainly been demonstrated in human muscle tissue. A handful of studies have examined age-related mitochondrial dysfunction in human blood cells, and only with small sample sizes and mainly in platelets. In this study, we analyzed mitochondrial respiration in peripheral blood mononuclear cells (PBMCs) and platelets from 308 individuals across the human lifespan (0–86 years). In regression analyses, with adjustment for false discovery rate (FDR), we found age-related changes in respiratory measurements to be either small or absent. The main significant changes were an age-related relative decline in complex I-linked respiration and a corresponding rise of complex II-linked respiration in PBMCs. These results add to the understanding of mitochondrial dysfunction in aging and to its possible role in immune cell and platelet senescence.

## Introduction

The role of mitochondria in aging has been recognized at least since the 1950s when the free radical theory of aging was first proposed^1^. While the causal relationship between free radicals and aging, as it was originally described, has been contradicted by more recent animal studies^2,3^, the complex association between mitochondria and aging has been consolidated and further explored^4^. Several other theories on how mitochondrial dysfunction may influence aging are currently being investigated. One such theory proposes that a gradual increase in mutations or deletions in the mitochondrial DNA (mtDNA) during the normal lifespan causes dysfunction in the electron transfer system (ETS), which leads to aging phenotypes^5,6^. This theory is supported by studies of knock-in mice with defective mtDNA polymerase^7^.

A gradual decline in mitochondrial respiration with age has been shown in human muscle tissue in several studies^8–11^. The magnitude of the decline is contested^12,13^; nonetheless, its potential link to sarcopenia, an important phenotype of frailty in aging, has been the focus of much research in recent years^14^.

Outside of muscle tissue, age-related mitochondrial dysfunction is far less studied, with differing profiles of declining function reported in human tissues such as the brain, liver, and intestine^15–17^. As for blood cells, only a handful of studies, with modest sample sizes and almost exclusively featuring platelets, have examined age-related mitochondrial decline, and the results have been inconclusive^18–23^. Hence, the question of mitochondrial function with aging in blood cells is still largely open. The aim of this study was to investigate the hypothesis that mitochondrial function declines with age in human PBMCs and platelets.

## Results

In 317 individuals aged 3 months to 86 years, mitochondrial function was analyzed separately in isolated PBMCs and isolated platelets. Nine participants with confirmed primary mitochondrial disease were excluded, and the remaining 308 individuals were analyzed for the effect of age on mitochondrial respiration and mitochondrial content. Data for 153 of the 308 participants have previously been published elsewhere in articles not focused on the effects of aging^24–30^. The study population comprised patient cohorts formed to study specific conditions, including patients with neurodegenerative disorders and sepsis, and healthy controls (for further details, see Methods). The patient cohorts consisted of 217 cases, and the remaining 91 were healthy controls (Table 1). Mitochondrial respiratory function was assessed by high-resolution respirometry using oxygraphy in intact and permeabilized cells (Supplementary Fig. 1, Supplementary Tables 1 and 2). These protocols allow for a comprehensive analysis of integrated function as well as separate pathways of electron transfer, i.e., through different enzyme complexes. In addition to routine (endogenous) respiration, measured in intact cells, maximum phosphorylating capacity (OXPHOS capacity), maximum electron transfer capacity (ET capacity), and the non-phosphorylating respiration needed to compensate for proton leak (leak respiration) were measured in permeabilized cells. Ratios of measurements were calculated to assess the relative contribution of CI-linked respiration (N/NS pathway control ratio; S = succinate, N = NADH), CII-linked respiration (S/NS pathway control ratio), and coupling efficiency (respiratory control ratio; RCR) (Supplementary Table 1).

**Table 1:** Study population.

	*n*	Female/male (not recorded)	Adults/children*	Age, median years (range)	Patients/healthy participants
Main study population	308	162/143 (3)	235/73	59 (0–86)	217/91
Population where CS activity was measured	159^a^	85/71 (3)	93/66	23 (0–84)	91/68

^a^CS was measured in both cell types in 157 cases. CS was measured only in platelets in two cases and only in PBMCs in one case.

**Fig. 1:**
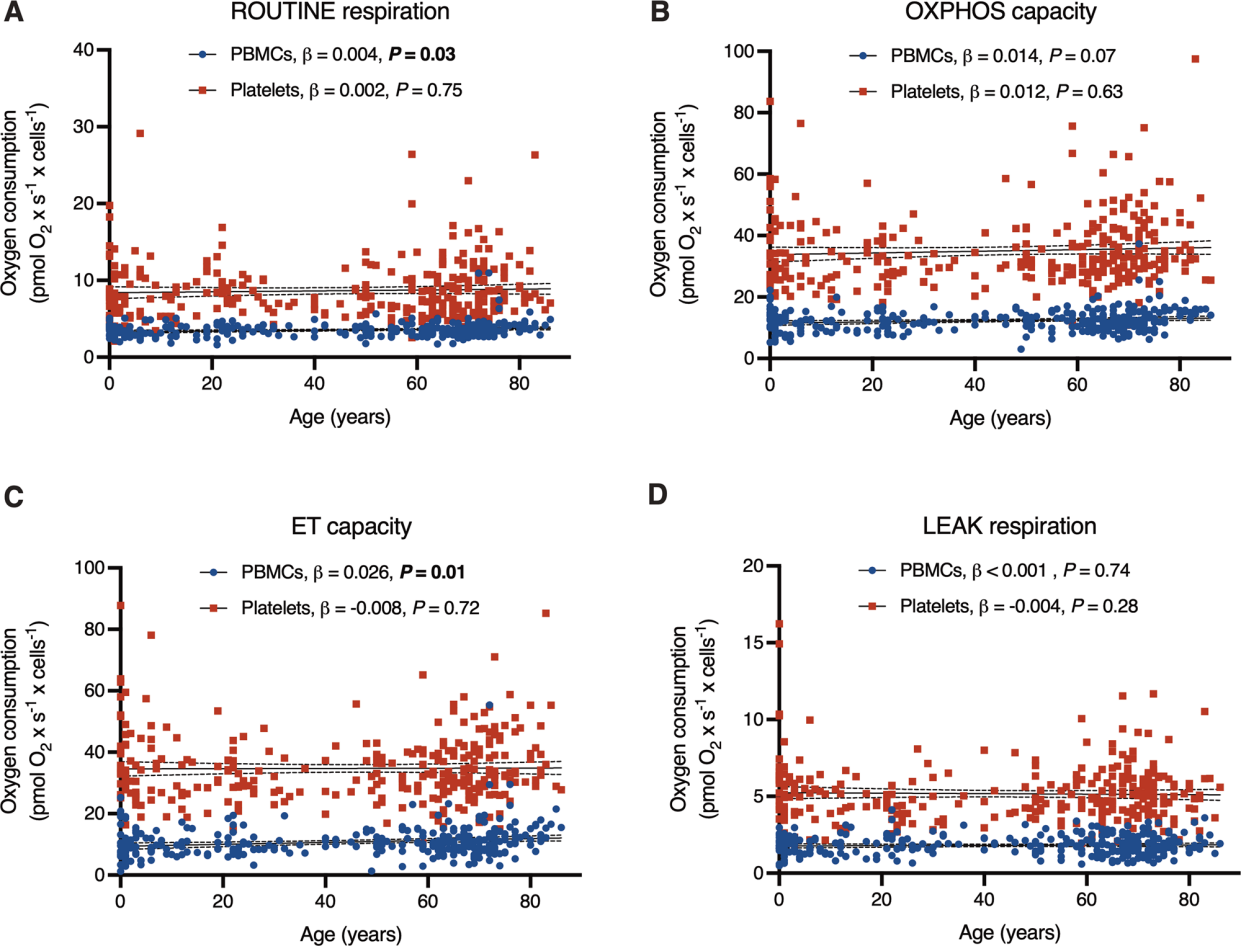
Cell count-normalized respiration as a function of age, with adjustment for confounders. (**A**–**D**) Plots depict simple linear regression for each respiratory parameter (*y*-axes) depending on age (*x*-axes); the slope is depicted as a straight line with its 95% confidence interval as a dotted line (complete data in Supplementary Table 3). The age effect, and its *P*-value, adjusted for sex and health status in a multiple regression (MR) model, is displayed in the respective graph legend. None of the age effects were classified as true discoveries after adjustment for the false discovery rate. The overall MR model was significant for routine respiration (*R*^2^ = 0.027, *P* = 0.04), OXPHOS capacity (*R*^2^ = 0.036, *P* = 0.01), and ET capacity (*R*^2^ = 0.047, 0.002) for PBMCs; none of the MR models were significant for platelets (complete MR data in Supplementary Table 4).

Unadjusted respiratory measurements normalized to cell count were plotted against age (Fig. 1, Supplementary Table 3). Multiple regression analyses were performed where sex and health status were used as binary covariates in addition to age for each respiratory parameter. The possibility of sex differences in mitochondrial respiration has garnered much attention and speculation, but a recent meta-analysis, including 2258 participants from 50 studies, demonstrated an overall lack of sex differences^31^. Therefore, sex differences were not expected to confound the age effect substantially. However, one of the few slight sex differences detected in the meta-analysis was a higher mitochondrial content in women’s leukocytes^31^. Allowing the possibility of confounding effects, sex was included as a covariate in all regression models. Since around two-thirds of the participants were selected from patient cohorts, and since several of these patients have diseases that may be associated with mitochondrial alterations, health status (patient cohort or healthy controls) was included as another covariate. The inclusion of the patient cohorts permitted a much greater sample size and higher statistical power. (simple regression for the healthy subgroup without adjustment for sex is provided in Supplementary Fig. 2, Supplementary Fig. 3, and Supplementary Table 9).

After adjusting for sex and health status, the only significant relationships in the respiratory parameters, normalized to cell count, were a slight age-dependent increase in routine respiration (slope = 0.004, SD ± 0.002, *P* = 0.03) and ET capacity (slope = 0.026, SD ± 0.010, *P* = 0.01) in PBMCs (Fig. 1, Supplementary Table 4). In platelets, none of the cell count-normalized respiratory measurements varied with age (Fig. 1, Supplementary Table 4). A notable fact is that the significant results in PBMCs showed an increase in respiration with advancing age, contrary to the hypothesis of a respiratory decline. However, the overall effect was slight, as the variation explained by the combined regression model accounted for under 5% of the total variation in respiration in the two significant parameters (*R*^2^ = 0.027 for routine respiration and *R*^2^ = 0.047 for ET capacity, respectively, in PBMCs). After calculating the false discovery rate (FDR) to control for the influence of multiple comparisons, these results were not classified as true discoveries.

**Fig. 2:**
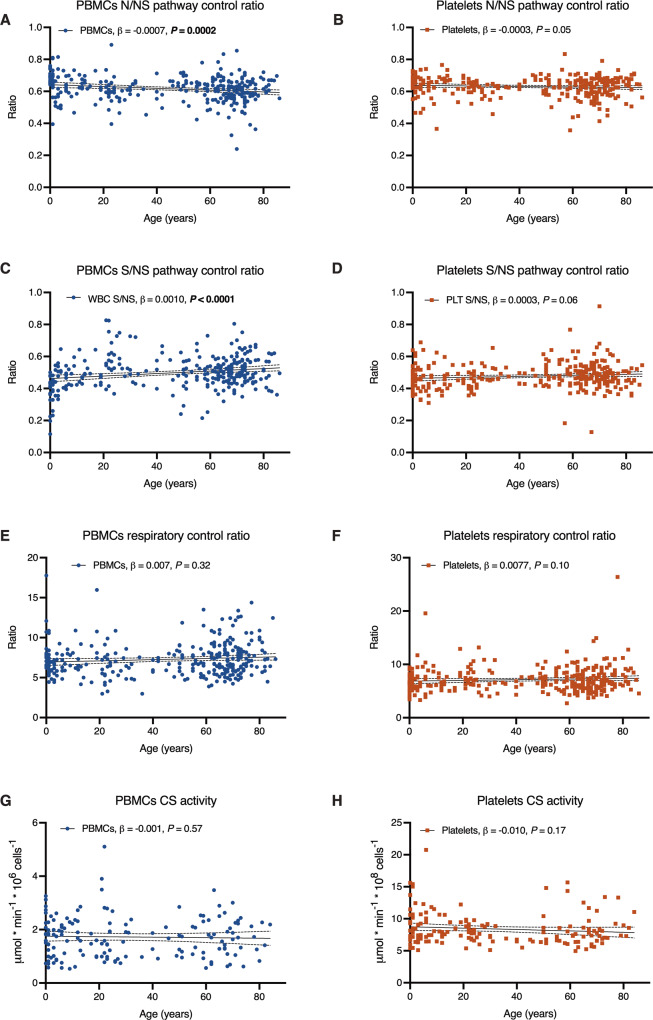
Ratios and CS activity as a function of age, with adjustment for confounders. Plots depict simple linear regression for each ratio (**A**–**F**) and citrate synthase (CS, **G**–**H**) activity (*y*-axes) depending on age (*x*-axes); the slope is depicted as a straight line with its 95% confidence interval as a dotted line (complete data in Supplementary Table 3). The age effect, and its *P*-value, adjusted for sex and health status in a multiple regression (MR) model, is displayed in the respective graph legend. Age effect *P*-values in bold were significant and classified as true discoveries after adjustment for false discovery rate. The overall MR model was significant for the N/NS pathway control ratio (*R*^2^ = 0.047, *P* = 0.002) and S/NS pathway control ratio (*R*^2^ = 0.086, *P* < 0.0001) for PBMCs; none of the MR models were significant for platelets (complete MR data in Supplementary Table 5).

The three respiratory ratios calculated from the respiratory measurements were plotted in the same manner as described above (Fig. 2, Supplementary Table 5). After adjusting for sex and health status, age-dependent changes remained highly significant in the N/NS pathway control ratio (slope = −0.0007, SD ± 0.0002, *P* = 0.0002) and the S/NS pathway control ratio for PBMCs (slope = 0.0010, SD ± 0.0002, *P* = < 0.0001) (Fig. 2, Supplementary Table 5). This can be summarized as a pattern of an age-dependent decrease in the relative contribution of CI-linked respiration, mirrored by an almost equal increase in the relative contribution of CII-linked respiration. Again, the explained variance was small for the combined model (*R*^2^ = 0.047 for N/NS pathway control ratio and R^2^ = 0.086 for S/NS pathway control ratio, respectively, in PBMCs) (Fig. 2, Supplementary Table 5), indicating that the variability in these parameters is mainly explained by factors other than age. The significant results for the age covariate and for the combined model for the N/NS and S/NS pathway control ratios were all classified as true discoveries after calculating FDR.

The RCR was not affected by age in PBMCs, and none of the ratios changed with age in platelets. CS activity (mitochondrial content) did not change with age in neither blood cell type. Also of note, after adjustment for age and health status, there were no significant sex differences in any parameter in neither PBMCs nor platelets, which is in line with recent evidence^31^.

**Fig. 3:**
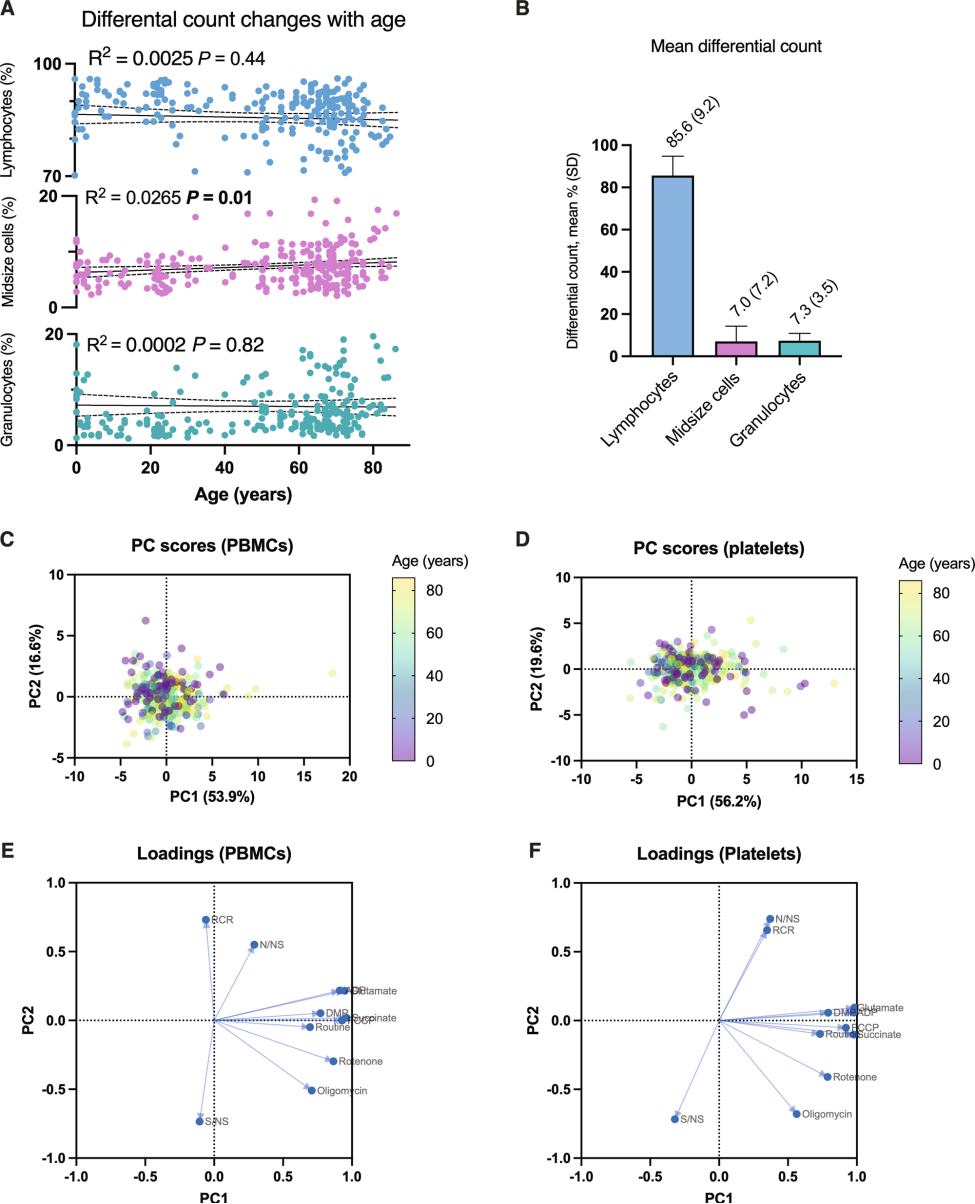
Differential count and principal component analysis. Linear regression of differential count (%) depending on age (**A**) for lymphocytes, midsize cells, and granulocytes (*n* = 262; slopes in Supplementary Table 6). 12, 1, and 8 data points, respectively, were removed from the lymphocyte, midsize cell, and granulocyte plots to compress the figure and enhance legibility. Mean differential count (%) and standard deviation (SD) for the same sample (**B**). Principal component (PC) scores for all respiratory measurements and derived ratios (Supplementary Tables 1 and 2) for PBMCS (**C**) and platelets (**D**) labeled by age. Corresponding loading plots for all parameters, for PBMCs (**E**) and platelets (**F**) (table of loadings in Supplementary Table 5).

Principal component analysis, using all respiratory rates and derived ratios in the regression analyses (Supplementary Table 1) and additional measurements from the study protocol (Supplementary Table 2) were performed. In the loadings plots (Fig. 3C, D), the measurements not selected for regression analysis had a relatively high level of collinearity with the ones used (bundled arrows pointing right in the graph), confirming that no important age-dependent respiratory change in the study protocol was overlooked when selecting the representative measurements for further analysis.

## Discussion

In summary, blood cells from a large human study population showed no age-dependent decline in mitochondrial respiration in platelets and only small changes in PBMCs. This is reflected in the PC score plots for PBMCs (Fig. 3C) and platelets (Fig. 3D), which did not reveal prominent clustering when the two first principal components were plotted with labeling for age. This finding runs contrary to, and nuances, the general notion of mitochondrial decline in aging but is not unique. As was previously touched upon, even the well-studied mitochondrial decline in skeletal muscle has turned out to be less evident than originally perceived. While several studies have found a general age-dependent decline in respiration in muscle mitochondria^8–11^, several comparable studies have found little or no decline^13,32,33^. One reason for the discrepancies may be methodological. Picard et al. found that the aging effects were much greater in isolated muscle mitochondria from rats when compared to mitochondria in permeabilized muscle bundles, suggesting that the isolation procedure itself more easily damages the frail (but otherwise functional) mitochondria of aged individuals^12^. A strength of the present study is that all experiments were made in both intact and permeabilized cells rather than in isolated mitochondria, more closely mimicking in vivo conditions.

One should also be cautious not to generalize the mitochondrial function and rate of decline across the entire organism, as these changes may be tissue-specific. Previous studies on human blood cells are the most relevant ones in comparison to the present study, but results from these have been inconsistent. Two studies found no age-dependent decline in platelet respiration^19,23^. Two other studies found a comparable decline in some respiratory parameters but contradictory results in others^21,22^. Differences in underlying study populations may account for the diverging results, as well as differences in statistical methods. The two latter studies compared group means and only one of the two former ones used a linear regression model. The statistical methods may have been limited by sample sizes, which were 27, 85, 59, and 64, respectively^19,21–23^. The sample size in the present study was 308, and the linear regression models allowed, in addition to examining the strength of the correlations, a quantification of the age effect. In a recent animal study with a large sample size, there was no decline in platelet respiratory function with age in 344 rats, even though such changes were seen in mitochondria from skeletal muscle and kidney cortex from the same rats^34^. We share the authors’ conclusion in that publication, further supported by the present findings, that one should be cautious not to generalize mitochondrial function across tissues.

To our knowledge, only one previous study has examined age-related decline in mitochondrial respiration in PBMCs. That study, in agreement with the present one, reported no decline in cell count-normalized respiration in a small human population (*n* = 38). However, when normalizing to mtDNA content, several parameters, such as routine respiration and ET capacity, did decline with age^19^. Although mtDNA was not analyzed for this study, another marker of mitochondrial content, CS activity, was analyzed for about half of the study population (Table 1). As a sensitivity analysis, the cell count-normalized respiratory parameters for PBMCs (Fig. 1, Supplementary Table 3) were normalized to CS activity and reanalyzed. Multiple regression analyses of this subset (*n* = 156) did not reveal an age-dependent decline for any parameter (Supplementary Table 8). It should be noted that even though CS activity and mtDNA are both widely used markers for mitochondrial content, their accuracy and mutual correlation vary in previous research^27,35^.

It is well known that blood differential count changes with age in humans^36^. The cells used in the PBMC experiments were isolated by a commercially available method, ensuring a high share of lymphocytes in the final suspension regardless of whole blood differential count. To make sure that the participants’ ages had not influenced the isolation process in a way that would have skewed the results, age-dependent variations in differential count were analyzed (for a majority of cases, *n* = 262). The proportion of lymphocytes did not change significantly depending on age, nor did the proportion of granulocytes (Fig. 3A). The proportion of midsize cells did slightly increase with age (Fig. 3A), but the explained variance was low (*R*^2^ = 0.027) and midsize cells, on average, only made up 7.0% (SD ± 7.2) of the analyzed PBMC samples (Fig. 3B). Taken together, it is unlikely that variations in PBMC composition could have influenced the results strongly in either direction, particularly in such a way that a large age-dependent respiratory decline would have gone undetected.

One should always be careful not to overinterpret negative results. Nonetheless, the findings of this study are interesting in relation to other advances in the understanding of blood cell senescence. While hematopoietic stem cell (HSC) function is known to decline with age, peripheral blood cells are continuously renewed from HSCs via progenitor cells in the bone marrow, and it is unclear at what level the age-related effects are determined^37^. Animal cell studies have described mechanisms by which stem cells can protect their progeny from damaged proteins, containing the effects of aging to the mother cell^38^. The immune system deterioration seen in aging (such as chronic inflammation and impaired immunity) may not be closely linked to the senescence of the individual peripheral cells. For example, in a recent study, T cells from mice were able to substantially outlive their species with retained ability to proliferate and fight infections^39^. Furthermore, it is known that the composition of peripheral lymphocyte subpopulations shifts with advancing age, and this could affect immune function through mechanisms unrelated to mitochondrial impairment or stem cell deterioration^40^.

As for platelets, it is known that they become hyperreactive with age, and this contributes to an increased risk for clotting and cardiovascular diseases^41^. The reason for this is not fully known, but the cause may lie outside of the platelets themselves, driven by dysregulated inflammatory pathways in the aged organism^41^. If that were the case, the altered function of platelets in aging would not need to be linked to a decline in aerobic respiratory function in the non-activated state, which was studied here.

A few significant age-dependent effects were observed in this study. The fact that CII-respiration increased relative to CI-respiration with age in PBMCs is of interest, even though the effect itself was small. Complex II differs from complex I in that it is completely determined by nuclear DNA, which in theory should make it unsusceptible to the mutations in mtDNA that are known to increase with age^5^. The CI–CII balance shift may represent compensation for a slight age-dependent decline in CI function that does not reach the threshold to influence overall metabolic or immune cell function.

There are several important limitations. The participants included are not representative of a healthy aging population and this could bias the results even though measures were taken to adjust for the influence of the patient cohorts. Another major limitation is the lack of data on several potential confounders, such as BMI, history of smoking, and exercise habits. While the age span of this study population is similar to the ones of many preceding studies of mitochondrial function and aging, the oldest participant was 86 years old, limiting the applicability of the results to mitochondrial function in advanced age (>80 years). Nonlinear relationships between aging and mitochondrial function were not examined; this decision was based on previous findings of linear relationships in other tissues.

In conclusion, this is, to date, the largest study on aging and mitochondrial respiration in human PBMCs and platelets. The results do not support a decline in respiratory function in peripheral blood cells during the normal human lifespan.

## Methods

### Participants

The study includes samples from 308 participants collected between 2008 and 2016 at Skåne University Hospital, Sweden, and Rigshospitalet, Denmark. They comprise all individuals (both patients and healthy controls) sampled for blood cell respirometry in our laboratory during that period, for which data for both PBMCs and platelets exist. Part of the data has been used in previous publications that have not focused on age-related effects on mitochondrial respiration^24–30^. The dataset includes both healthy controls and patients. The latter group includes patients with neurodegenerative movement disorders and with sepsis (sampled at the time of admittance) and a group of pediatric patients with diffuse complaints seen at a pediatric A&E department. Patients with known primary mitochondrial disease were not included.

The included projects were subject to approval by the regional ethical review board of Lund University, Sweden (No 2008/113, 2009/97, 2011/89, and 2011/320) and the scientific ethical committee of Copenhagen County, Denmark (H-C-2008-023) and all participants, or guardian/parent or next of kin, as stipulated by the respective ethical permit, signed informed consent.

### Sample preparation

Participants were sampled through a venous puncture or through an existing arterial line and peripheral blood drawn into K_2_EDTA tubes. Samples were analyzed within 3–5 hours. Cell concentrations in whole blood and cell suspensions were measured using a Swelab Alfa automated hemocytometer (Swelab, Stockholm, Sweden).

Cell isolation was performed at room temperature. Erythrocytes and leukocytes were loosely pelleted by centrifugation at 300–400*g* for 10–15 min, leaving a platelet-rich plasma (PRP). The pellet was resuspended in saline, and lymphocytes were isolated using Lymphoprep (axis-shield PoC AS). The resuspended cells were layered on top of the Lymphoprep and centrifuged at 800*g* for 20–30 min. The resulting lymphocyte layer was pipetted off and resuspended in saline, and centrifuged at 250*g* for 5 min. After removing the supernatant, the lymphocyte pellet was resuspended in 100–200 µL saline. The final suspension contained 20–30% plasma. The lymphocyte suspension contained up to 30% granulocytes and midsize cells (monocytes, eosinophils, basophils, etc.).

The PRP (from the initial centrifugation) was collected and centrifuged for 5 minutes at 4 600*g*, producing close to cell-free plasma and a platelet pellet. The platelet pellet was resuspended in 1–3 mL of the participant’s own plasma by gentle pipetting to obtain a highly enriched PRP.

### High-resolution respirometry

Measurement of mitochondrial respiration was performed in an oxygraph (Oxygraph-2k, Oroboros Instruments, Innsbruck, Austria) at a constant temperature of 37 °C. Platelets were suspended at a concentration of 200 × 10^6^/ml, except for six cases where there was insufficient sample (primarily for pediatric participants, conc. 19, 126, 152, 154, 190, and 190 × 10^6^ platelets/mL, respectively). PBMCs were suspended at a concentration of 2.5–5 × 10^6^/ml, as the sample amount allowed. Calibration with air-saturated Millipore water was performed daily. A mitochondrial respiration medium (MiR05) containing sucrose 110 mM, HEPES 20 mM, taurine 20 mM, K-lactobionate 60 mM, MgCl_2_ 3 mM, KH_2_PO_4_ 10 mM, EGTA 0.5 mM, BSA 1 g/l, pH 7.1 was used for all experiments. Oxygen solubility factors relative to pure water were set to 0.92, and stirrer speed to 200. Data were recorded in DatLab version 4.3 (Oroboros Instruments, Innsbruck, Austria).

A schematic description and a representative trace of a standard experiment are shown in Supplementary Fig. 1, and the respiratory parameters measured in or derived from the experiment are listed in Supplementary Tables 1 and 2.

Routine respiration was measured in intact platelets with endogenous substrates. Cells were permeabilized using digitonin (1 µg per 10^6^ platelets or 6 µg per 10^6^ PBMCs), and simultaneously, the N-pathway substrates (or CI substrates) malate and pyruvate were added at saturating concentrations (5 mM each). Subsequently, adenosine diphosphate (ADP) was added (1 mM), followed by another N-pathway substrate, glutamate (5 mM). This was followed by S-pathway substrate (CII substrate) succinate (10 mM) to reach maximum phosphorylating capacity through both N- and S-pathways (both CI and CII). This respiration rate is labeled OXPHOS capacity (alternatively known as NS-OXPHOS capacity).

Next, the ATP synthase was inhibited by oligomycin (1 µg/ml), revealing LEAK respiration. This was followed by the titration of carbonyl cyanide-p-tri-fluoromethoxyphenylhydrazone (FCCP) to induce maximal capacity of the electron transport system (ETS) in the uncoupled state, labeled ET capacity (alternatively known as NS-ET capacity). This was followed by the inhibition of CI by rotenone (2 µM) to measure the S-pathway (CII) in the uncoupled state. After this, the CIII-inhibitor antimycin A (1 µg/ml) was added to halt electron transfer through the ETS altogether, and the residual oxygen consumption (Rox) measured at this point was subtracted from all other measurements before any further analyses were made.

The N/NS pathway control ratio (N/NS PCR) reflects the relative contribution of the N-pathway or CI-linked respiration. The S/NS-pathway control ratio (S/NS PCR) reflects the relative contribution of the S-pathway or CII-linked respiration. The RCR reflects the coupling efficiency of the ETS. In contrast to the other respiratory measurements, which were normalized to cell count, respiratory ratios are a way of normalizing results to mitochondrial content, providing qualitative indices of mitochondrial function. Supplementary Table 1 describes how each ratio was calculated.

### Citrate synthase activity

After completion of the respirometry, the content of each oxygraph chamber (2 ml) was stored at −80 °C. A subset of the samples was later thawed for analysis of CS activity using a commercial kit (Citrate Synthase Assay Kit, CS0720, Sigma-Aldrich, St Louis, MO, USA). Before the enzyme assay, the thawed samples were sonicated on ice for 30 s for PBMCs, and 2 sets of 30 s for platelets (Ultrasonic homogenizer 4710 Series; Cole-Parmer Instrument Company LLC, Vernon Hills, IL). The assay was performed in accordance with the manufacturer’s instructions, as previously described^29^.

### Statistical methods

All statistical analyses were performed, and all data figures were created using PRISM GraphPad version 9.1.2 (La Jolla, USA) unless otherwise specified. Four measurements of respiratory function and three ratios of respiratory rates were selected based on previous literature in order to cover relevant aspects of ETS function. Together with CS activity, a marker of mitochondrial content, there were eight parameters selected for the main analysis. These parameters were plotted against age in scatterplots with a straight line illustrating the slope of simple linear regression and dotted lines to illustrate its 95% confidence interval. Multiple regressions were performed for each parameter, adjusting the results for sex (coded as females = 0, males = 1) and health status (coded as healthy cohort = 0, disease cohort = 1).

Principal component analyses (PCA) were made separately for PBMCs and platelets using a correlation matrix and including all parameters used in the regression analysis (Supplementary Table 1), as well as additional parameters measured in the study protocol (Supplementary Table 2). Data were not transformed prior to ordination. The principal components were selected using parallel Monte Carlo analysis with 1000 simulations and a percentile level of 95%. The purpose of the PCA was to illustrate the overall effect of age on respiratory parameters available for selection and to analyze collinearity to control for unintended omission of important age-dependent parameters.

To further control for possible sources of error, a differential count was performed in the PBMC suspension after the lymphocyte isolation procedure. Linear regressions were made to see whether differential count varied across age. Mean cell contamination of platelets in the PBMC samples, and vice versa, was calculated in a subset of the samples.

For all regression analyses described above, a *P*-value < 0.05 was considered statistically significant at the first stage of interpretation. To control for multiple comparisons, a false discovery rate (FDR) analysis was performed using the adaptive linear step-up procedures with Q set to 5 on all 112 *P*-values from both the linear (Figs. 1 and 2; Supplementary Tables 3 and 8) and the multiple (Supplementary Tables 4, 5, and 8) regressions^42^.

### Reporting summary

Further information on research design is available in the Nature Research Reporting Summary linked to this article.

### Supplementary information

Supplementary material

Reporting summary

## Data Availability

The data that support the findings of this study are available from the corresponding author, J.E., upon reasonable request.
