# Supplementary material for: Mitochondrial function in peripheral blood cells across the human lifespan
Source: NPJ Aging. 2024 Feb 7;10(1):10. doi: 10.1038/s41514-023-00130-4 (PMC10850142; doi:10.1038/s41514-023-00130-4)
Supplement: Supplementary file 1 — Supplementary material [file 41514_2023_130_MOESM1_ESM.pdf]

Supplementary Tables and Figures

Supplementary Table 1: Parameters used in regression analyses

Name	Abbreviation	Corresponding measurement or calculation	Explanation
ROUTINE respiration	*R*	DMP*	Routine respiration in intact cells with endogenous substrates.
OXPHOS capacity	$PGMS_P$	succinate*	Maximal ADP-stimulated respiration with pyruvate, malate, glutamate and succinate (NS pathway/CI-CII-linked pathway).
ET capacity	$PGMS_E$	FCCP*	Maximal noncoupled respiration with pyruvate, malate, glutamate and succinate (NS pathway/CI-CII-linked pathway).
LEAK respiration	*L*(Omy)	oligomycin*	Background respiration (with oligomycin-inhibited ATP synthase) with pyruvate, malate, glutamate and succinate. Consists mainly of proton leak over the inner mitochondrial membrane.
N/NS pathway control ratio	N/NS PCR	glutamate/succinate	Relative N-pathway/CI-linked pathway function (in the coupled state)
S/NS pathway control ratio	S/NS PCR	rotenone/FCCP	Relative S-pathway/CII-linked pathway function (in the noncoupled state)
Respiratory control ratio	RCR	succinate/oligomycin	Coupling efficiency (OPXHOS capacity relative LEAK respiration)
Citrate synthase activity	CS activity	*Separate enzyme assay, see Methods section.*	An enzyme marker for mitochondrial content.

The abbreviations listed are used in this publication and/or are used in reference literature. The third column indicates after which addition to the chamber respiration was measured or which combination of measurements were used to create the respiratory ratios, these correspond to Figure 1. P = OXPHOS; L = LEAK; E = ET; N = NADH ; S = succinate; CI = complex I; CII = complex II; CIV = complex IV; DMP = digitonin, malate, pyruvate; PM = pyruvate, malate; PGM = pyruvate, glutamate, malate; PGMS = pyruvate, glutamate, malate, succinate; FCCP = protonophore carbonyl cyanide 4-(trifluoromethoxy) phenylhydrazone; ADP = adenosine triphosphate *Corrected for residual oxygen consumption (Rox) measured after the addition of antimycin A

Supplementary Table 2: Additional parameters included in the principal component analysis

Name	Abbreviation	Corresponding measurement or calculation	Explanation
LEAK respiration (without adenylates)	L(n)	DMP*	Leak respiration in permeabilized cells with malate and pyruvate in the absence of exogenous ADP. Sometimes referred to as *Background respiration*.
N-OXPHOS capacity (with pyruvate and malate)	PM_P	ADP*	ADP-stimulated respiration with malate and pyruvate, without glutamate.
N-OXPHOS capacity	PGM_P	glutamate*	ADP-stimulated respiration with malate, pyruvate and glutamate (N-pathway/CI-linked respiration)
S-ET capacity	$PGMS_{E+Rot}$	rotenone*	Non-coupled respiration with malate, pyruvate and glutamate and complex I-inhibitor Rotenone (S-pathway/CII-linked respiration)

Columns and abbreviations are explained in the Supplementary Table 1 legend. *Corrected for residual oxygen consumption (Rox) measured after the addition of antimycin A

Supplementary Table 3: Simple linear regression, respiration rates, ratios and CS depending on age

	PBMCs			Platelets		
	R^2	Slope	*P* value	R^2	Slope	*P* value
ROUTINE respiration	0.023	0.0052	**0.008**	0.002	-0.0058	0.43
OXPHOS capacity	0.022	0.0188	**0.009**	0.004	0.0268	0.24
ET capacity	0.031	0.0313	**0.002***	<0.001	0.0035	0.88
LEAK respiration	0.001	0.0008	0.55	0.001	-0.0016	0.67
N/NS PCR	0.037	-0.0006	**0.0007***	0.006	-0.0002	0.19
S/NS PCR	0.047	0.0008	**0.0001***	0.013	0.0003	0.05
RCR	0.011	0.0076	0.07	0.006	0.0062	0.19
CS activity	0.001	-0.0009	0.68	0.014	-0.0107	0.14

The left column shows the dependent variables. The first four parameters correspond to the graphs in Figure 1 and are measured in pmol O_2 * s^{-1} * 10^6 $cells^{-1}$ for PBMCs and pmol O_2 * s^{-1} * 10^8 $cells^{-1}$ for platelets. The latter four parameters correspond to Figure 2; the first three are ratios of respiratory measurements and the last, CS activity, is measured in μmol * min^{-1} * 10^6 $cells^{-1}$ for PBMCs and μmol * min^{-1} * 10^8 $cells^{-1}$ for platelets. All regressions are calculated with age in years as the independent variable. Sample size is 308 for all parameters in both cell types except RCR (which was 307 for PBMCs) and CS (which was 156 for PBMCs and 158 for platelets). PC = pathway control ratio; RCR = respiratory control ratio; CS = citrate synthase *True discovery after false discovery rate-analysis

Supplementary Table 4: Multiple regression, cell count-normalized respiration depending on age

**Respiration rate**	Routine respiration	OXPHOS capacity	ET capacity	LEAK respiration
PBMCs				
**Model summary**				
R^2	0.027	0.036	0.047	0.002
DF	301	301	301	301
F-ratio	2.86	3.75	4.92	0.24
P	**0.04**	**0.01**	**0.002***	0.87
Constant	3.200	10.94	8.296	1.74
**Independent variables**				
Age				
Slope (SE)	0.004 (0.002)	0.014 (0.007)	0.026 (0.010)	<0.001 (0.001)
P	**0.03**	0.07	**0.01**	0.74
Sex				
Slope (SE)	0.023 (0.112)	0.186 (0.402)	0.996 (0.565)	0.035 (0.075)
P	0.83	0.64	0.08	0.64
Disease				
Slope (SE)	0.175 (0.127)	1.039 (0.455)	1.185 (0.641)	0.048 (0.085)
P	0.17	**0.02**	0.07	0.57
Platelets				
**Model summary**				
R^2	0.008	0.020	0.018	0.023
DF	301	301	301	301
F-ratio	0.812	2.05	1.82	2.36
P	0.49	0.11	0.14	0.07
Constant	8.442	32.00	32.28	4.91
**Independent variables**				
Age				
Slope (SE)	0.002 (0.008)	0.012 (0.024)	-0.008 (0.023)	-0.004 (0.004)
P	0.75	0.63	0.72	0.28
Sex				
Slope (SE)	-0.400 (0.417)	0.418 (1.293)	1.578 (1.249)	0.016 (0.212)
P	0.34	0.75	0.21	0.94
Disease				
Slope (SE)	0.477 (0.473)	3.284 (1.465)	2.859 (1.415)	0.6282 (0.240)
P	0.32	**0.03**	**0.04**	**0.01**

Multiple regression analyses calculated to predict respiration rate (Routine respiration, NS-OXPHOS capacity, NS-ET capacity and LEAK respiration, measured in pmol $O^2 * s^{-1} * 10^6$ cells^{-1} for PBMCs and $O^2 * s^{-1} * 10^8$ cells^{-1} for platelets) based on age (years), sex (coded as female=0, male=1) and disease state (coded as healthy control=0, participant from a patient cohort=1). P values for significant regression models and for significant predictors ($p<0.05$) written in bold. R^2 = explained variance; DF = degrees of freedom; SE = standard error; *True discovery after false discovery rate-analysis.

Supplementary Table 5: Multiple regression, respiratory ratios and CS depending on age

**Respiration rate**	N/NS pathway control ratio (CI-linked)	S/NS pathway control ratio (CII-linked)	Respiratory control ratio	CS activity
PBMCs				
**Model summary**				
R^2	0.047	0.086	0.005	0.038
DF	301	301	301	149
F-ratio	4.93	9.48	0.47	1.94
p	**0.002***	**<0.0001***	0.71	0.13
Constant	0.634	0.47	6.71	1.778
**Independent variables**				
Age				
Slope (SE)	-0.0007 (0.0002)	0.0010 (0.0002)	0.007 (0.007)	-0.001 (0.002)
P	**0.0002***	**<0.0001***	0.32	0.57
Sex				
Slope (SE)	0.005 (0.009)	0.018 (0.011)	0.204 (0.364)	-0.179 (0.124)
P	0.62	0.10	0.57	0.15
Disease				
Slope (SE)	0.014 (0.010)	-0.037 (0.013)	0.064 (0.412)	0.204 (0.124)
P	0.19	**0.004***	0.88	0.10
Platelets				
**Model summary**				
R^2	0.020	0.024	0.009	0.058
DF	301	301	301	151
F-ratio	2.05	2.48	0.90	3.12
P	0.11	0.06	0.44	**0.03**
Constant	0.632	0.450	6.88	8.34
**Independent variables**				
Age				
Slope (SE)	-0.0003 (0.0001)	0.0003 (0.0002)	0.0077 (0.0047)	-0.010 (0.007)
P	0.05	0.06	0.10	0.17
Sex				
Slope (SE)	-0.001 (0.007)	0.0123 (0.0088)	-0.0056 (0.2544)	-0.163 (0.394)
P	0.87	0.16	0.98	0.68
Disease				
Slope (SE)	-0.016 (0.008)	0.0102 (0.0100)	-0.1286 (0.2884)	1.004 (0.393)
P	0.05	0.30	0.66	**0.01**

Multiple regression analyses calculated to predict respiratory ratios (N/NS pathway control ratio, S/NS pathway control ratio and respiratory control ratio), and CS activity (μmol * min^{-1} * 10^6 $cells^{-1}$ for PBMCs, μmol * min^{-1} * 10^8 $cells^{-1}$ for platelets), based on age (years), sex (coded as female=0, male=1) and disease state (coded as healthy control=0, participant from a patient cohort=1). P values for significant regression models and for significant predictors (p<0.05) written in bold. CS = citrate synthase; R^2 = explained variance; DF = degrees of freedom; SE = standard error; *True discovery after false discovery rate-analysis.

Supplementary Table 6: Loadings for principal component analyses

	PBMCs			Platelets	
**Principal component** (eigenvalue)	**PC1** (5.933)	**PC2** (1.823)	**PC3** (1.226)	**PC1** (6.180)	**PC2** (2.160)
Routine	0.695	-0.049	0.281	0.732	-0.098
DMP	0.771	0.052	0.253	0.791	0.056
ADP	0.911	0.217	-0.118	0.977	0.058
Glutamate	0.947	0.215	-0.118	0.982	0.093
Succinate	0.960	0.019	0.106	0.977	-0.102
Oligomycin	0.708	-0.510	-0.401	0.563	-0.680
FCCP	0.928	-0.001	0.105	0.920	-0.053
Rotenone	0.865	-0.297	0.183	0.787	-0.410
N/NS PCR	0.291	0.550	-0.598	0.371	0.738
S/NS PCR	-0.106	-0.735	0.307	-0.322	-0.718
RCR	-0.061	0.731	0.622	0.348	0.656

Number of principal components retained for each blood cell type was determined by parallel analysis, as described in **Methods**. Eigenvalues (lower-upper limits) from parallel analysis were, for PBMCs, PC1: 1.311 (1.245-1.397), PC2: 1.223 (1.170-1.282), PC3: 1.155 (1.110-1.202); and for platelets, PC1: 1.306 (1.240-1.392), PC2 1.220 (1.170-1.280). For abbreviations and explanations of variables, see **Supplementary Table 1 and 2**.

Supplementary Table 7: Simple linear regression, differential count depending on age (years)

	n	R^2	Slope	*P* value
Lymphocytes (%)	262	0.0025	0.0230	0.44
Granulocytes (%)	262	0.0002	-0.0040	0.82
Midsize cells (%)	262	0.0265	0.0086	**0.01**

Corresponds to graph shown in Figure 3A.

Supplementary Table 8: Multiple regression, CS-normalized respiration depending on age in PBMCS

Respiration rate	Routine respiration	OXPHOS capacity	ET capacity	LEAK respiration
PBMCs (n=156)				
**Model summary**				
R^2	0.06	0.007	0.028	0.008
DF	152	152	152	152
F-ratio	1.311	1.352	2.475	1.429
P	0.27	0.26	0.06	0.24
Constant	2.576	8.53	7.41	1.37
**Independent variables**				
Age				
Slope (SE)	0.002 (0.004)	0.013 (0.012)	0.011 (0.010)	0.002 (0.002)
P	0.60	0.26	0.29	0.42
Sex				
Slope (SE)	-0.238 (0.203)	-0.656 (0.638)	-1.217 (0.559)	-0.142 (0.121)
P	0.24	0.31	**0.03**	0.18
Disease				
Slope (SE)	-0.277 (0.203)	-0.735 (0.637)	-0.550 (0.559)	-0.161 (0.120)
P	0.18	0.25	0.33	0.18

Multiple regression analyses calculated to predict respiration rate normalized to CS-activity, measured in pmol $O^2 * s^{-1} * ml^{-1} * IU^{-1}$, based on age (years), sex (coded as female=0, male=1) and disease state (coded as healthy control=0, participant from a patient cohort=1). R^2 = explained variance; DF = degrees of freedom; SE = standard error (Analyses in this table were performed by SPSS version 28.0.0.0, IBM Corp., Armonk, NY.)

Supplementary Table 9: Simple linear regression. Respiration rates, ratios and CS depending on age

	PBMCs			Platelets		
	R^2	Slope	*P* value	R^2	Slope	*P* value
ROUTINE respiration	0.002	0.0009	0.71	0.002	0.0046	0.67
OXPHOS capacity	0.021	0.0144	0.17	0.004	0.0197	0.56
ET capacity	0.033	0.0211	0.08	0.001	-0.0114	0.74
LEAK respiration	0.004	-0.0016	0.53	0.007	0.0043	0.45
N/NS PCR	<0.001	0.0001	0.86	0.023	-0.0003	0.15
S/NS PCR	0.025	0.0006	0.14	0.058	0.0006	**0.02**
RCR	0.060	0.0205	**0.02**	0.005	-0.0061	0.52
CS activity	0.005	-0.0023	0.52	0.102	-0.0228	**0.003***

The left column shows the dependent variables. The first four parameters correspond to the graphs in Supplementary Figure 2 and are measured in pmol O_2 * s^{-1} * 10^6 $cells^{-1}$ for PBMCs and pmol O_2 * s^{-1} * 10^8 $cells^{-1}$ for platelets. The latter four parameters correspond to Supplementary Figure 3; the first three are ratios of respiratory measurements and the last, CS activty, is measured in μmol * min^{-1} * 10^6 $cells^{-1}$ for PBMCs and μmol * min^{-1} * 10^8 $cells^{-1}$ for platelets. All regressions are calculated with age in years as the independent variable. Sample size is 91 for all parameters for CS activity, were n=83 (for both cell types). PC = pathway control ratio; RCR = respiratory control ratio; CS = citrate synthase; *True discovery after false discovery rate-analysis.

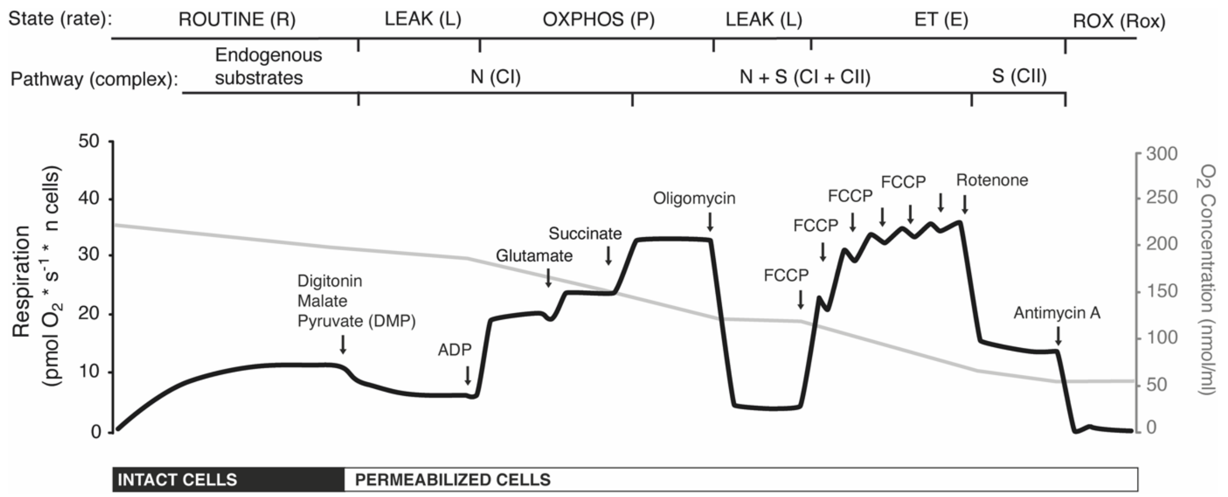

Supplementary Figure 1: Schematic illustration of the study protocol. The gray line describes the O_2 concentration in the chamber (right y-axis) and the black line describes O_2 flux normalized to 10^8 platelets or 10^6 PBMCs (left y-axis). The arrows mark additions of substrates, inhibitors or uncouplers (and, in one case, digitonin). The first top bar indicates respiratory states (and corresponding rates). The second top bar indicates the pathway (or respiratory complex) through which electrons are donates. CI = complex I; CII = complex II; N = NADH-linked substrates; S = succinate; OXPHOS = oxidative phosphorylation; ET = electron transfer; ROX = residual oxygen consumption

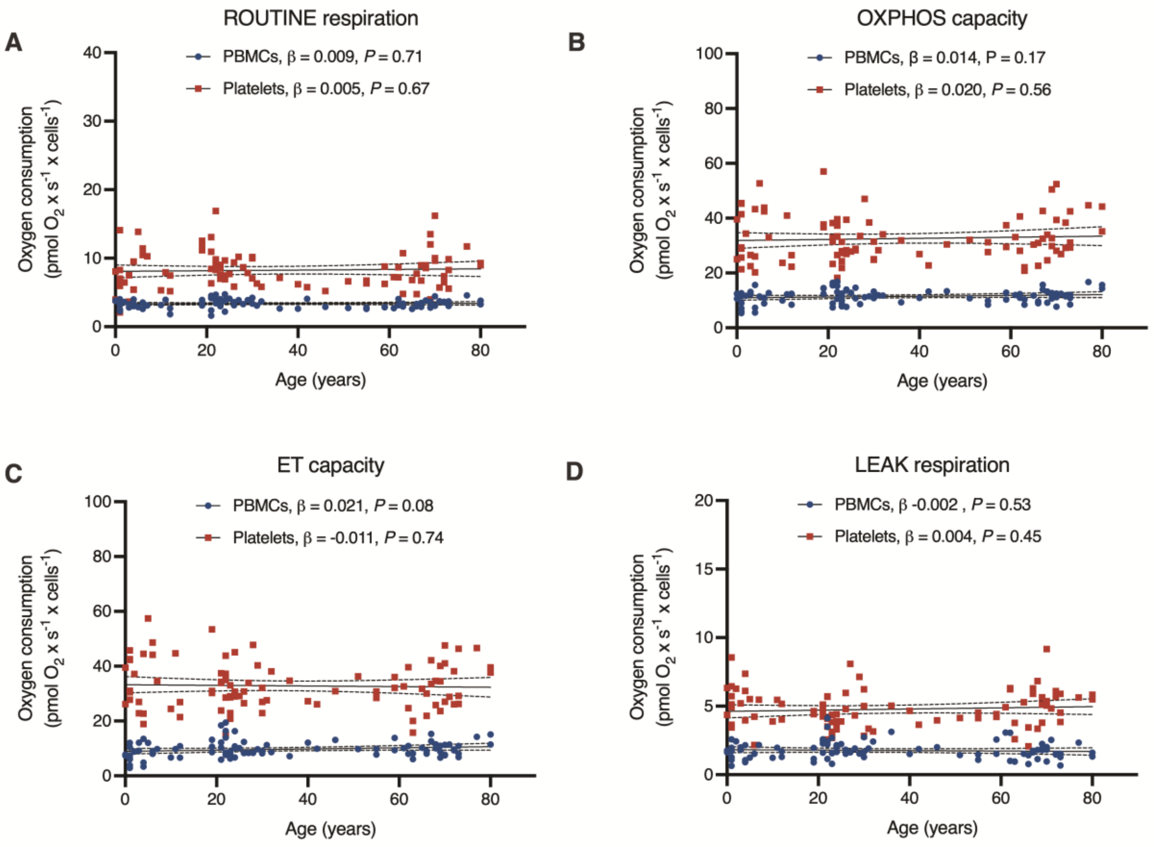

Supplementary Figure 2. Cell count-normalised respiration as a function of age in healthy participants Plots depict simple linear regression for each respiratory parameter (y-axes) depending on age (x-axes), the slope depicted as a straight line with its 95% confidence interval as a dotted line (complete data in **Supplementary Table 9**).

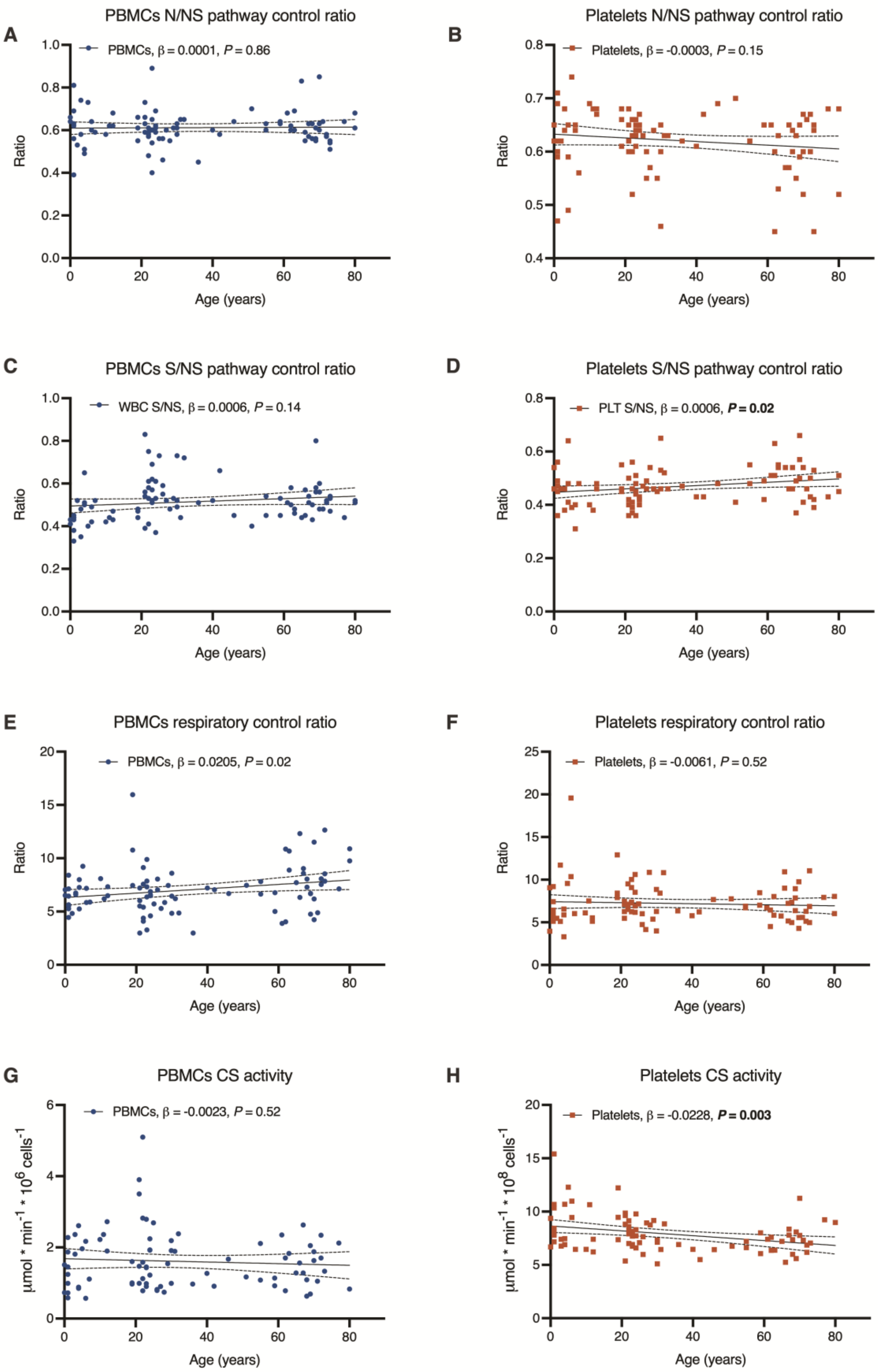

Supplementary Figure 3. Ratios and CS activity as a function of age in healthy participants. CS = citrate synthase. Plots depict simple linear regression for each ratio and CS activity (y-axes) depending on age (x-axes), the slope depicted as a straight line with its 95% confidence interval as a dotted line (complete data in **Supplementary Table 9**).
